# Prediction of hemodynamic severity of coarctation: a magnetic resonance imaging based prediction tree

**DOI:** 10.1186/1532-429X-13-S1-P197

**Published:** 2011-02-02

**Authors:** Stefano Muzzarelli, Jeffery J Meadows, Karen Gomes Ordovas, Michael D Hope, Charles B Higgins, James C Nielsen, Tal Geva, Alison Knauth Meadows

**Affiliations:** 1 University Hospital Basel, Basel, Switzerland; 2 UCSF, San Francisco, CA, USA; 3 Children’s Hospital Boston, Boston, MA, USA

## Objectives

To create a cardiovascular magnetic resonance (CMR) based tree algorithm for predicting coarctation (CoA) transcatheter systolic pressure gradient > 20mmHg.

## Background

A published algorithm containing minimal aortic cross-section area and the flow deceleration pattern in the descending aorta measurements obtained by CMR can predict the presence of severe CoA (systolic pressure gradient at catheterization of > 20mmHg). However, this algorithm has not been validated by another institution, and as a result its applicability to other centers has been uncertain. We sought to: 1. Assess the diagnostic accuracy of the existing algorithm for prediction of severe CoA using an external data source; 2. Refine the prediction model utilizing additional patients from two institutions; and 3. Create a clinically practical prediction tree based on cutoff values.

## Methods

Seventy-nine consecutive patients who underwent both CMR and cardiac catheterization for evaluation of native or recurrent CoA at the Children’s Hospital of Boston (CHB, n=30) and the University of California San Francisco (UCSF, n=49) were retrospectively reviewed. The published algorithm derived exclusively from data obtained at CHB was first validated from data obtained at UCSF, and diagnostic characteristics were determined. Next, pooled data from both institutions were analyzed and a refined model was created using logistic regression methods. Finally, recursive partitioning was used to develop a clinical prediction tree focused upon best fit of sensitivity and specificity at predicting severe CoA.

## Results

Severe CoA was present in 48 patients (60%). Indexed minimal aortic cross-sectional area (OR=0.63 per 10 mm/m^2^ increase, 95%-CI: 0.47-0.85) and heart rate-corrected flow deceleration time in the descending aorta (OR=1.22 per 0.01 s ^0.5^ increase, 95%-CI: 1.08-1.38) were independent predictors of CoA gradient > 20mmHg (p<0.01 for both). A clinical prediction tree combining these variables reached a sensitivity and specificity of 90% and 76%, respectively (figure).

**Figure 1:**
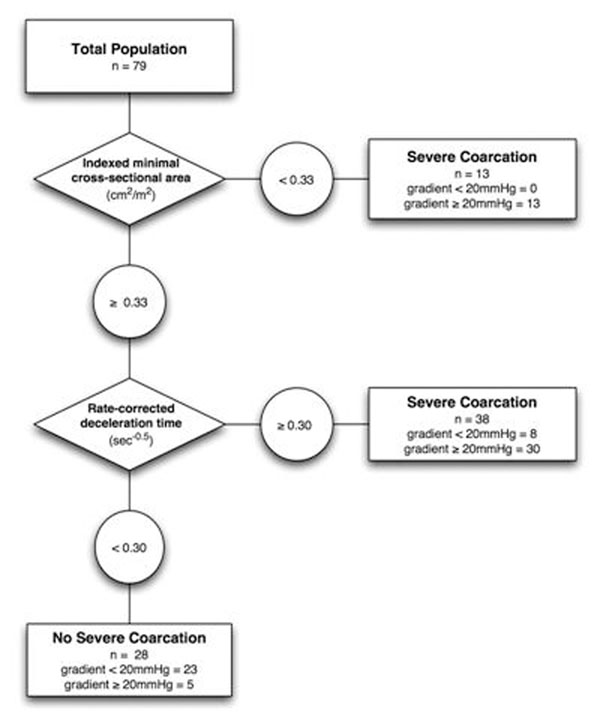


## Conclusion

CMR derived minimal aortic cross-sectional area and heart rate-corrected flow deceleration time in the descending aorta predict CoA gradient > 20 mmHg at subsequent catheterization. The presented prediction tree based on cutoff values is easy to use in clinical practice and may help to guide further diagnostic and therapeutic management of patients investigated for CoA.

